# Tetra-*n*-propyl­ammonium acetate–boric acid (1/1)

**DOI:** 10.1107/S1600536809044225

**Published:** 2009-10-31

**Authors:** Yun-Xia Yang, Qi Li, Seik Weng Ng

**Affiliations:** a College of Chemistry, Beijing Normal University, Beijing 100875, People’s Republic of China; b Department of Chemistry, University of Malaya, 50603 Kuala Lumpur, Malaysia

## Abstract

In the crystal structure of the ammonium carboxyl­ate–boric acid cocrystal, (C_3_H_7_)_4_N^+^·CH_3_CO_2_^−^·H_3_BO_3_, the boric acid forms two O—H⋯O hydrogen bonds to the acetate anion. The acetate–boric acid species is hydrogen bonded to another acetate–boric acid species through the third OH unit of the boric acid about a twofold rotation axis.

## Related literature

For the crystal structure of tetra-*n*-propyl penta­borate–boric acid co-crystal, see: Freyhardt *et al.* (1994 ▶).
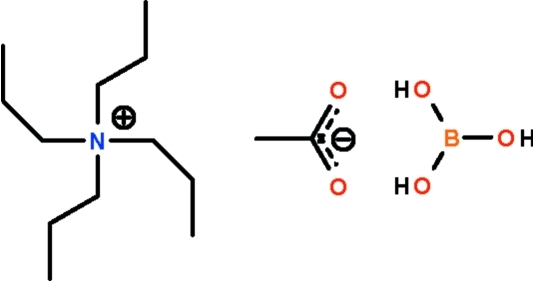


## Experimental

### 

#### Crystal data

C_12_H_28_N^+^·C_2_H_3_O_2_^−^·BH_3_O_3_
*M* *_r_* = 307.23
Orthorhombic, 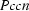

*a* = 16.4594 (3) Å
*b* = 16.7680 (3) Å
*c* = 14.4526 (3) Å
*V* = 3988.79 (13) Å^3^
*Z* = 8
Mo *K*α radiation
μ = 0.07 mm^−1^
*T* = 293 K
0.24 × 0.24 × 0.22 mm

#### Data collection

Bruker APEXII diffractometer
Absorption correction: none
19907 measured reflections
4581 independent reflections
2803 reflections with *I* > 2σ(*I*)
*R*_int_ = 0.022

#### Refinement

*R*[*F* ^2^ > 2σ(*F* ^2^)] = 0.051
*wR*(*F* ^2^) = 0.183
*S* = 1.02
4581 reflections
204 parameters
3 restraints
H atoms treated by a mixture of independent and constrained refinement
Δρ_max_ = 0.23 e Å^−3^
Δρ_min_ = −0.13 e Å^−3^

### 

Data collection: *APEX2* (Bruker, 2007 ▶); cell refinement: *SAINT* (Bruker, 2007 ▶); data reduction: *SAINT*; program(s) used to solve structure: *SHELXS97* (Sheldrick, 2008 ▶); program(s) used to refine structure: *SHELXL97* (Sheldrick, 2008 ▶); molecular graphics: *X-SEED* (Barbour, 2001 ▶); software used to prepare material for publication: *publCIF* (Westrip, 2009 ▶).

## Supplementary Material

Crystal structure: contains datablocks global, I. DOI: 10.1107/S1600536809044225/xu2651sup1.cif

Structure factors: contains datablocks I. DOI: 10.1107/S1600536809044225/xu2651Isup2.hkl

Additional supplementary materials:  crystallographic information; 3D view; checkCIF report

## Figures and Tables

**Table 1:** Hydrogen-bond geometry (Å, °)

*D*—H⋯*A*	*D*—H	H⋯*A*	*D*⋯*A*	*D*—H⋯*A*
O1—H1⋯O4	0.86 (1)	1.75 (1)	2.600 (2)	175 (2)
O2—H2⋯O5	0.85 (1)	1.79 (1)	2.638 (2)	172 (3)
O3—H3⋯O1^i^	0.85 (1)	1.88 (1)	2.729 (2)	174 (2)

Symmetry code: (i) 
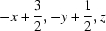
.

**Fig. 1.**
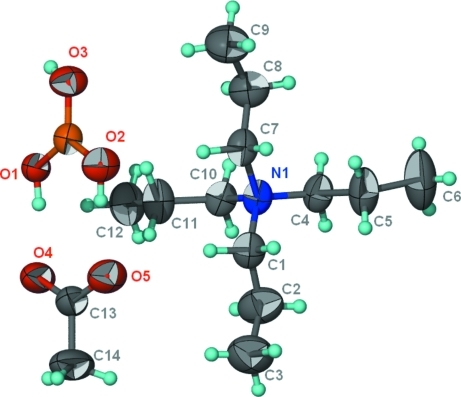
Thermal ellipsoid plot (Barbour, 2001) of [N(C3H7)4] [CH3CO2].H3BO3 at the 50% probability level; hydrogen atoms are drawn as spheres of arbitrary radius.

## supplementary crystallographic information

### Experimental

1,3,5-Tris(4-carboxyphenyl)benzene (0.055 g, 0.125 mmol) and boric acid (0.25 mmol, 0.015 g) were dissolved in a water-ethanol (50/100 *v*/*v*) mixture. An aqueous solution of 30% tetra-*n*-propylammonium hydroxide was added in an acid:base ratio of 1.3. Several drops of acetic acid were added and the clear solution set aside for the formation of crystals after several weeks.

### Refinement

Carbon-bound H-atoms were placed in calculated positions (C—H 0.93–0.97 Å) and were included in the refinement in the riding model approximation, with *U*(H) set to 1.2–1.5*U*(C). The hydroxy H-atoms were located in a difference Fourier map and were refined with a distance restraint of 0.85±0.01 Å; their temperature factors were freely refined.

### Crystal data

**Table:**

C_12_H_28_N^+^·C_2_H_3_O_2_^−^·BH_3_O_3_	*F*(000) = 1360
*M**_r_* = 307.23	*D*_x_ = 1.023 Mg m^−^^3^
Orthorhombic, *P**c**c**n*	Mo *K*α radiation, λ = 0.71073 Å
Hall symbol: -P 2ab 2ac	Cell parameters from 5166 reflections
*a* = 16.4594 (3) Å	θ = 2.2–25.7°
*b* = 16.7680 (3) Å	µ = 0.07 mm^−^^1^
*c* = 14.4526 (3) Å	*T* = 293 K
*V* = 3988.79 (13) Å^3^	Block, colorless
*Z* = 8	0.24 × 0.24 × 0.22 mm

### Data collection

**Table:**

Bruker APEXII diffractometer	2803 reflections with *I* > 2σ(*I*)
Radiation source: fine-focus sealed tube	*R*_int_ = 0.022
graphite	θ_max_ = 27.5°, θ_min_ = 2.2°
ω and ω scans	*h* = −20→21
19907 measured reflections	*k* = −21→21
4581 independent reflections	*l* = −18→17

### Refinement

**Table:**

Refinement on *F*^2^	Secondary atom site location: difference Fourier map
Least-squares matrix: full	Hydrogen site location: inferred from neighbouring sites
*R*[*F*^2^ > 2σ(*F*^2^)] = 0.051	H atoms treated by a mixture of independent and constrained refinement
*wR*(*F*^2^) = 0.183	*w* = 1/[σ^2^(*F*_o_^2^) + (0.0888*P*)^2^ + 0.6196*P*] where *P* = (*F*_o_^2^ + 2*F*_c_^2^)/3
*S* = 1.02	(Δ/σ)_max_ = 0.001
4581 reflections	Δρ_max_ = 0.23 e Å^−^^3^
204 parameters	Δρ_min_ = −0.13 e Å^−^^3^
3 restraints	Extinction correction: *SHELXL97* (Sheldrick, 2008), Fc^*^=kFc[1+0.001xFc^2^λ^3^/sin(2θ)]^-1/4^
Primary atom site location: structure-invariant direct methods	Extinction coefficient: 0.0029 (8)

### Fractional atomic coordinates and isotropic or equivalent isotropic displacement parameters (Å^2^)

**Table:**

	*x*	*y*	*z*	*U*_iso_*/*U*_eq_	
O1	0.65031 (7)	0.26154 (7)	0.38659 (8)	0.0628 (3)	
H1	0.5997 (7)	0.2504 (13)	0.3918 (13)	0.083 (6)*	
O2	0.60919 (9)	0.39766 (8)	0.38631 (11)	0.0822 (4)	
H2	0.5628 (10)	0.3765 (17)	0.396 (2)	0.131 (11)*	
O3	0.74569 (8)	0.36496 (8)	0.37814 (10)	0.0783 (4)	
H3	0.7800 (12)	0.3273 (10)	0.3834 (16)	0.104 (8)*	
O4	0.49799 (8)	0.22127 (8)	0.39542 (12)	0.0942 (5)	
O5	0.46002 (8)	0.34620 (9)	0.41629 (12)	0.0923 (5)	
N1	0.57206 (9)	0.42369 (8)	0.68832 (9)	0.0628 (4)	
C1	0.48754 (11)	0.42868 (11)	0.64651 (13)	0.0732 (5)	
H1A	0.4724	0.4845	0.6421	0.088*	
H1B	0.4898	0.4076	0.5840	0.088*	
C2	0.42181 (13)	0.38518 (18)	0.69837 (19)	0.1086 (8)	
H2A	0.4354	0.3290	0.7024	0.130*	
H2B	0.4179	0.4061	0.7608	0.130*	
C3	0.34159 (15)	0.39487 (19)	0.6499 (2)	0.1243 (10)	
H3A	0.3001	0.3675	0.6841	0.186*	
H3B	0.3452	0.3728	0.5888	0.186*	
H3C	0.3283	0.4505	0.6459	0.186*	
C4	0.57384 (13)	0.45737 (12)	0.78584 (12)	0.0750 (5)	
H4A	0.5387	0.4250	0.8245	0.090*	
H4B	0.6287	0.4522	0.8097	0.090*	
C5	0.54799 (17)	0.54338 (15)	0.79533 (16)	0.1045 (8)	
H5A	0.5782	0.5761	0.7520	0.125*	
H5B	0.4907	0.5484	0.7809	0.125*	
C6	0.5634 (2)	0.5719 (2)	0.8927 (2)	0.1579 (16)	
H6A	0.5471	0.6266	0.8982	0.237*	
H6B	0.6203	0.5672	0.9066	0.237*	
H6C	0.5327	0.5399	0.9353	0.237*	
C7	0.62782 (12)	0.46947 (10)	0.62404 (12)	0.0693 (5)	
H7A	0.6252	0.4450	0.5633	0.083*	
H7B	0.6072	0.5234	0.6182	0.083*	
C8	0.71601 (13)	0.47402 (14)	0.65287 (17)	0.0922 (6)	
H8A	0.7204	0.5036	0.7103	0.111*	
H8B	0.7367	0.4206	0.6635	0.111*	
C9	0.76609 (17)	0.51429 (15)	0.5795 (2)	0.1162 (9)	
H9A	0.8216	0.5173	0.5995	0.174*	
H9B	0.7456	0.5671	0.5690	0.174*	
H9C	0.7630	0.4842	0.5231	0.174*	
C10	0.59924 (11)	0.33770 (10)	0.69796 (12)	0.0647 (4)	
H10A	0.6521	0.3372	0.7276	0.078*	
H10B	0.5616	0.3106	0.7390	0.078*	
C11	0.60502 (16)	0.29084 (12)	0.61010 (14)	0.0875 (7)	
H11A	0.6441	0.3158	0.5691	0.105*	
H11B	0.5527	0.2907	0.5793	0.105*	
C12	0.63080 (17)	0.20624 (12)	0.62980 (17)	0.0967 (7)	
H12A	0.6343	0.1771	0.5727	0.145*	
H12B	0.5916	0.1813	0.6696	0.145*	
H12C	0.6829	0.2064	0.6596	0.145*	
C13	0.44575 (11)	0.27397 (13)	0.40662 (15)	0.0781 (5)	
C14	0.35937 (14)	0.24694 (18)	0.4140 (3)	0.1411 (12)	
H14A	0.3351	0.2698	0.4682	0.212*	
H14B	0.3577	0.1898	0.4183	0.212*	
H14C	0.3299	0.2639	0.3601	0.212*	
B1	0.66733 (12)	0.34081 (11)	0.38298 (13)	0.0591 (5)	

### Atomic displacement parameters (Å^2^)

**Table:**

	*U*^11^	*U*^22^	*U*^33^	*U*^12^	*U*^13^	*U*^23^
O1	0.0555 (7)	0.0561 (7)	0.0769 (8)	0.0019 (5)	0.0024 (5)	−0.0096 (5)
O2	0.0748 (9)	0.0586 (7)	0.1131 (12)	0.0115 (6)	0.0009 (8)	0.0088 (7)
O3	0.0670 (8)	0.0588 (7)	0.1091 (11)	−0.0027 (6)	0.0061 (7)	0.0111 (7)
O4	0.0616 (8)	0.0783 (9)	0.1426 (14)	−0.0003 (6)	0.0061 (8)	−0.0330 (8)
O5	0.0673 (8)	0.0771 (9)	0.1325 (13)	0.0137 (7)	0.0019 (8)	−0.0022 (8)
N1	0.0729 (8)	0.0629 (8)	0.0527 (8)	0.0194 (6)	−0.0066 (6)	0.0040 (6)
C1	0.0755 (12)	0.0766 (12)	0.0675 (11)	0.0210 (9)	−0.0140 (9)	0.0027 (9)
C2	0.0780 (14)	0.137 (2)	0.1105 (19)	0.0113 (14)	−0.0109 (13)	0.0199 (16)
C3	0.0839 (16)	0.134 (2)	0.155 (3)	0.0127 (15)	−0.0238 (16)	−0.0001 (19)
C4	0.0852 (12)	0.0848 (12)	0.0548 (10)	0.0224 (10)	−0.0081 (9)	−0.0031 (9)
C5	0.133 (2)	0.0994 (16)	0.0814 (14)	0.0463 (15)	−0.0204 (13)	−0.0260 (12)
C6	0.199 (4)	0.165 (3)	0.109 (2)	0.071 (3)	−0.046 (2)	−0.063 (2)
C7	0.0914 (12)	0.0551 (9)	0.0613 (10)	0.0137 (9)	−0.0014 (9)	0.0079 (7)
C8	0.0873 (14)	0.0955 (15)	0.0937 (15)	0.0014 (11)	−0.0033 (11)	0.0164 (12)
C9	0.114 (2)	0.1079 (18)	0.127 (2)	−0.0218 (15)	0.0101 (16)	0.0175 (16)
C10	0.0757 (11)	0.0598 (9)	0.0584 (10)	0.0148 (8)	−0.0033 (8)	0.0128 (7)
C11	0.1289 (19)	0.0639 (11)	0.0698 (12)	0.0226 (11)	−0.0088 (12)	0.0032 (9)
C12	0.1239 (18)	0.0607 (11)	0.1054 (17)	0.0151 (11)	−0.0040 (14)	−0.0001 (11)
C13	0.0555 (10)	0.0852 (14)	0.0936 (14)	0.0044 (9)	−0.0024 (9)	−0.0198 (11)
C14	0.0603 (13)	0.125 (2)	0.238 (4)	−0.0056 (14)	0.0121 (17)	−0.054 (2)
B1	0.0658 (11)	0.0570 (10)	0.0545 (10)	0.0053 (8)	0.0001 (8)	0.0027 (8)

### Geometric parameters (Å, °)

**Table:**

O1—B1	1.359 (2)	C5—H5B	0.9700
O1—H1	0.856 (9)	C6—H6A	0.9600
O2—B1	1.352 (2)	C6—H6B	0.9600
O2—H2	0.853 (10)	C6—H6C	0.9600
O3—B1	1.354 (2)	C7—C8	1.512 (3)
O3—H3	0.851 (9)	C7—H7A	0.9700
O4—C13	1.244 (2)	C7—H7B	0.9700
O5—C13	1.242 (2)	C8—C9	1.503 (3)
N1—C7	1.515 (2)	C8—H8A	0.9700
N1—C10	1.516 (2)	C8—H8B	0.9700
N1—C4	1.519 (2)	C9—H9A	0.9600
N1—C1	1.519 (2)	C9—H9B	0.9600
C1—C2	1.505 (3)	C9—H9C	0.9600
C1—H1A	0.9700	C10—C11	1.496 (3)
C1—H1B	0.9700	C10—H10A	0.9700
C2—C3	1.503 (3)	C10—H10B	0.9700
C2—H2A	0.9700	C11—C12	1.508 (3)
C2—H2B	0.9700	C11—H11A	0.9700
C3—H3A	0.9600	C11—H11B	0.9700
C3—H3B	0.9600	C12—H12A	0.9600
C3—H3C	0.9600	C12—H12B	0.9600
C4—C5	1.510 (3)	C12—H12C	0.9600
C4—H4A	0.9700	C13—C14	1.496 (3)
C4—H4B	0.9700	C14—H14A	0.9600
C5—C6	1.508 (3)	C14—H14B	0.9600
C5—H5A	0.9700	C14—H14C	0.9600
			
B1—O1—H1	114.6 (15)	N1—C7—H7A	108.3
B1—O2—H2	110 (2)	C8—C7—H7B	108.3
B1—O3—H3	113.9 (16)	N1—C7—H7B	108.3
C7—N1—C10	111.08 (13)	H7A—C7—H7B	107.4
C7—N1—C4	111.66 (15)	C9—C8—C7	110.8 (2)
C10—N1—C4	105.23 (12)	C9—C8—H8A	109.5
C7—N1—C1	106.44 (13)	C7—C8—H8A	109.5
C10—N1—C1	111.05 (14)	C9—C8—H8B	109.5
C4—N1—C1	111.49 (13)	C7—C8—H8B	109.5
C2—C1—N1	115.70 (16)	H8A—C8—H8B	108.1
C2—C1—H1A	108.4	C8—C9—H9A	109.5
N1—C1—H1A	108.4	C8—C9—H9B	109.5
C2—C1—H1B	108.4	H9A—C9—H9B	109.5
N1—C1—H1B	108.4	C8—C9—H9C	109.5
H1A—C1—H1B	107.4	H9A—C9—H9C	109.5
C3—C2—C1	110.3 (2)	H9B—C9—H9C	109.5
C3—C2—H2A	109.6	C11—C10—N1	116.12 (13)
C1—C2—H2A	109.6	C11—C10—H10A	108.3
C3—C2—H2B	109.6	N1—C10—H10A	108.3
C1—C2—H2B	109.6	C11—C10—H10B	108.3
H2A—C2—H2B	108.1	N1—C10—H10B	108.3
C2—C3—H3A	109.5	H10A—C10—H10B	107.4
C2—C3—H3B	109.5	C10—C11—C12	110.60 (16)
H3A—C3—H3B	109.5	C10—C11—H11A	109.5
C2—C3—H3C	109.5	C12—C11—H11A	109.5
H3A—C3—H3C	109.5	C10—C11—H11B	109.5
H3B—C3—H3C	109.5	C12—C11—H11B	109.5
C5—C4—N1	115.72 (15)	H11A—C11—H11B	108.1
C5—C4—H4A	108.4	C11—C12—H12A	109.5
N1—C4—H4A	108.4	C11—C12—H12B	109.5
C5—C4—H4B	108.4	H12A—C12—H12B	109.5
N1—C4—H4B	108.4	C11—C12—H12C	109.5
H4A—C4—H4B	107.4	H12A—C12—H12C	109.5
C6—C5—C4	109.9 (2)	H12B—C12—H12C	109.5
C6—C5—H5A	109.7	O5—C13—O4	125.24 (18)
C4—C5—H5A	109.7	O5—C13—C14	117.86 (19)
C6—C5—H5B	109.7	O4—C13—C14	116.8 (2)
C4—C5—H5B	109.7	C13—C14—H14A	109.5
H5A—C5—H5B	108.2	C13—C14—H14B	109.5
C5—C6—H6A	109.5	H14A—C14—H14B	109.5
C5—C6—H6B	109.5	C13—C14—H14C	109.5
H6A—C6—H6B	109.5	H14A—C14—H14C	109.5
C5—C6—H6C	109.5	H14B—C14—H14C	109.5
H6A—C6—H6C	109.5	O2—B1—O3	117.74 (16)
H6B—C6—H6C	109.5	O2—B1—O1	122.85 (17)
C8—C7—N1	115.99 (15)	O3—B1—O1	119.39 (15)
C8—C7—H7A	108.3		
			
C7—N1—C1—C2	178.63 (18)	C10—N1—C7—C8	−60.1 (2)
C10—N1—C1—C2	57.6 (2)	C4—N1—C7—C8	57.0 (2)
C4—N1—C1—C2	−59.4 (2)	C1—N1—C7—C8	178.90 (16)
N1—C1—C2—C3	180.0 (2)	N1—C7—C8—C9	174.83 (18)
C7—N1—C4—C5	60.1 (2)	C7—N1—C10—C11	−57.4 (2)
C10—N1—C4—C5	−179.33 (19)	C4—N1—C10—C11	−178.41 (18)
C1—N1—C4—C5	−58.9 (2)	C1—N1—C10—C11	60.8 (2)
N1—C4—C5—C6	−172.4 (2)	N1—C10—C11—C12	−178.76 (18)

### Hydrogen-bond geometry (Å, °)

**Table:**

*D*—H···*A*	*D*—H	H···*A*	*D*···*A*	*D*—H···*A*
O1—H1···O4	0.86 (1)	1.75 (1)	2.600 (2)	175 (2)
O2—H2···O5	0.85 (1)	1.79 (1)	2.638 (2)	172 (3)
O3—H3···O1^i^	0.85 (1)	1.88 (1)	2.729 (2)	174 (2)

Symmetry codes: (i) −*x*+3/2, −*y*+1/2, *z*.
